# A Rare Case of Hodgkin Lymphoma With Isolated Epitrochlear Lymphadenopathy. Our Experience and Main Differential Diagnosis

**DOI:** 10.1155/carm/5548125

**Published:** 2025-07-10

**Authors:** Laura Cea, Domiziana Santucci, Caterina Bernetti, Lorenzo Nibid, Valeria Tomarchio, Amalia Bruno, Bruno Beomonte Zobel, Rosario Francesco Grasso, Eliodoro Faiella

**Affiliations:** ^1^Department of Radiology, Fondazione Policlinico Universitario Campus Bio-Medico, Via Alvaro Del Portillo, Rome 21-00128, Italy; ^2^Department of Anatomo-Pathology, Fondazione Policlinico Universitario Campus Bio-Medico, Via Alvaro Del Portillo, Rome 21-00128, Italy; ^3^Department of Hematology, Fondazione Policlinico Universitario Campus Bio-Medico, Via Alvaro Del Portillo, Rome 21-00128, Italy

**Keywords:** anatomical pathology, diagnostic imaging, elbow, epitrochlear lymphadenopathy, hematology, Hodgkin lymphoma, soft tissue tumor

## Abstract

Hodgkin lymphoma (HL) is a type of lymphoma, characterized by the presence of abnormal Reed-Sternberg cells. It typically affects lymph nodes, generally in the upper body (such as neck, chest, and armpits). It can also involve the spleen, liver, and bone marrow. In the literature, there are sporadic cases of atypical localization of HL. The aim of this article is to report a peculiar case of HL in a 55-year-old male presenting with primary epitrochlear lymphadenopathy as the only localization of disease, also performing a literature review on this atypical presentation. We also summarized the possible underlying malignant pathologies that arise from the soft tissues of the upper limb adjacent to the elbow.

## 1. Introduction

Hodgkin lymphoma (HL) is a type of lymphoma that accounts for approximately 10% of cases of newly diagnosed lymphoma [1]. The most common onset symptom of HL is swelling, generally not painful, of a lymph node located in the neck, trunk, axilla, or in the inguinal region, in association with systemic symptoms such as fever, night sweats, and weight loss [2]. Even though the involvement of other lymph nodes, such as the epitrochlear or popliteal ones, is rare [3], over the years, a few authors have documented cases of HL manifesting primarily in the epitrochlear region, as a local manifestation of a systemic disease [4] or as an isolated presentation [5]. This peculiar presentation, if properly managed—mainly through radiotherapy (RT) and/or chemotherapy (CT)—exhibits a prognosis similar to that of HL occurring in a more typical location. Nevertheless, the available literature on this topic remains extremely scarce. Lesions arising from soft tissues of the upper limb may be benign or malignant. The most common malignant lesions include synovial sarcoma, myxofibrosarcoma, undifferentiated pleomorphic sarcoma, and malignant peripheral nerve sheath tumors (MPNSTs).

The aim of this article is to report a peculiar case of HL presenting with primary epitrochlear lymphadenopathy, and to perform a literature review of malignant lesions that arise from the soft tissues of the upper limb adjacent to the elbow, providing a quick differential diagnosis.

## 2. Case Report

We present a case study involving a 55-year-old-male, who came to our attention in April 2020 during an interventional radiology examination, in order to assess a tumefaction (swelling) on the right elbow, causing pain in the arm flexion. The lesion had been present for nearly 2 years with a slight noticeable growth in the last few months.

During physical examination, a palpable, solid, moderately hard mass was detected in the medial and distal portion of the right arm, without associated skin alterations.

No additional anomalies were observed during the physical examination, including redness, temperature elevation, or signs of infection, as well as systemic symptoms of lymphoma (“B symptoms” such as fever, night sweats, and unintentional weight loss) and no relevant past medical or surgical history was declared.

Laboratory investigations were promptly carried out, reporting normal haematological and biochemical values, without significant alteration in the blood count and a lymphocyte/neutrophil ratio and leukocyte count within normal ranges.

**Figure 1:**
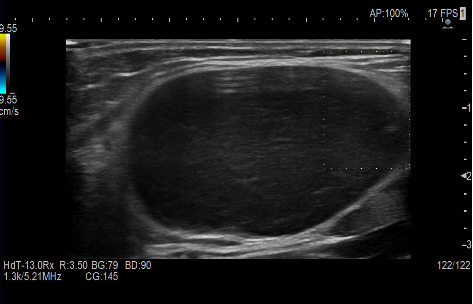
US exam. US images at the level of the palpable lesion demonstrating an oval, well defined, capsulated, hypoechoic mass of 4 cm, with a minimal peripheral signal at color Doppler imaging, indicating the enlarged epitrochlear lymph node with loss of the normal hyperechoic hilar structure.

**Figure 2:**
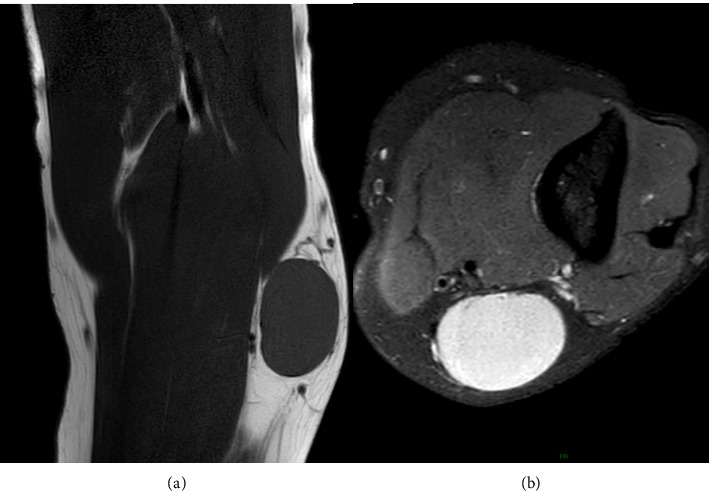
MRI exam. Coronal T1 TSE MRI sequence (a) demonstrating a hypointense, occupying space mass, localized in the upper para-epitroclear seat, that in the axial T2 TSE MRI sequence (b) appear homogeneously hyperintense.

At the location of the palpated mass, ultrasound (US) examination demonstrated a 4 cm oval, well defined, capsulated, hypoechoic mass with a minimal peripheral signal at Color Doppler imaging (Figure 1), potentially indicating an enlarged lymph node with loss of the normal hyperechoic hilar structure. The subsequent diagnostic investigation through Magnetic Resonance Imaging (MRI) showed an occupying space lesion at the medial region of the distal third of the arm, in the upper para-epitrochlear seat, characterized by thin pseudocapsule and some thin septa. On T1-weighted sequence the lesion presented with low signal, whereas appeared homogeneously hyperintense on T2-weighted fat-suppressed (FS) sequences (Figure 2).

Given the MRI and US imaging features and the growth over time, a needle biopsy was indicated. The procedure was performed under local anesthesia, in a sterile field, under US-guidance, using coaxial techniques to avoid seeding.

Histopathological examination revealed the presence of lymphatic tissue, consisting mainly of small lymphocytes, with presence of germinal centers, a small portion of eosinophils and some thin fibrotic striae. Large neoplastic cells were observed with one or more voluminous nuclei characterized by prominent eosinophilic nucleoli (Hodgkin and Reed Sternberg cells) and in which the immunohistochemical investigation documents the following phenotype: CD30+, CD15+, weak Pax5+, CD20−, CD3−, MUM1+, LMP1−, EBER-ISH−, ALK−, CD45−.

**Figure 3:**
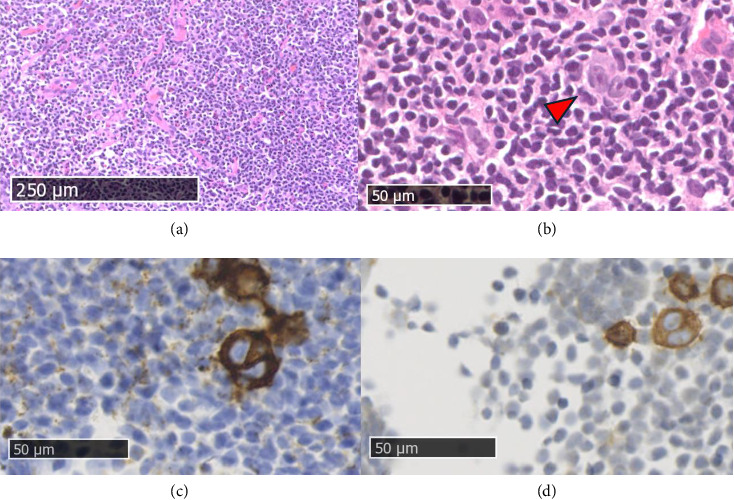
Histological sample. (a) The tumor cells were scattered among abundant nonatypical lymphocytes (HE, × 100). (b) Close-up on Reed-Sternberg cells (red arrowhead, HE, × 400). (c) (d) CD30 and CD15 positive Hodgkin and Reed-Sternberg cells (HE, × 400).

Histological findings were referable to a classic-type HL, lymphocyte-rich type, according to WHO 2017 (Clinical stage CS IA, pathological stage PS IA) (Figure 3).

The patient was HIV negative, with a negative family history for lymphoma and for other risk factors.

No additional palpable lymph node was detected on the physical examination, in particular in ipsilateral axillary cable; no clinical B symptoms, such as fever and weight loss within a short period with an unknown reason; and no night sweating, itching, cough, respiratory distress, or chest pain was detected. No hepatosplenomegaly was determined.

**Figure 4:**
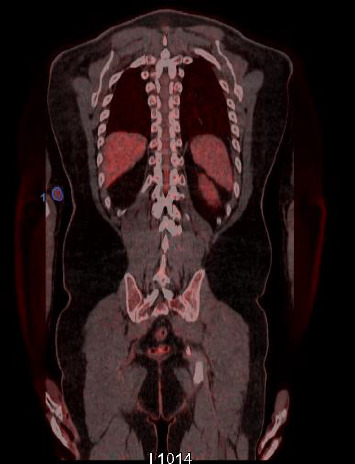
PET exam. Tracer distribution images (335 Mbq 18F-FDG) detected pathological uptake only at the level of the right epitrochlear lymph node region.

In the histopathological examination of the bone marrow biopsy, no finding in favor of lymphoma was detected. The results were compatible with normocellular bone marrow. The postoperative blood tests, alanine aminotransferase, aspartate aminotransferase, lactate dehydrogenase, C-reactive protein, protein electrophoresis, erythrocyte sedimentation rate, and fibrinogen values were within normal ranges. The patient, who underwent clinical staging, was examined by fluorodeoxyglucose/positron emission tomography-computed tomography (FDG/PET-CT) imaging for a correct staging. PET-CT scan demonstrated a slightly increased FDG uptake at the region of the epitrochlear lymph node of the right arm. No FDG uptake except the right epitrochlear lymph node region was detected (Figure 4). The patient underwent two courses of CT (CT following ABVD protocol-doxorubicin, bleomycin, vinblastine, and dacarbazine) plus RT with the diagnosis of lymphocyte-rich-type HL (CS IA). At the end of the treatment, the patient performed US and PET follow-up, with 3 year disease-free controls, without any complication.

## 3. Discussion

Epitrochlear lymph nodes, usually in number of one, two, rarely three or four, are located on the medial face of the elbow and are component of the superficial lymphatic system of the upper limb [6].

Physical examination, as well as patient anamnesis, routine blood tests, and US examination, are fundamental in the investigation of masses within the elbow region [6].

Selby et al., in a study, examined the epitrochlear region of 140 healthy individuals compared with 185 patients affected by diseases presenting with lymphadenopathy [7]. The aim of the study was to identify the presence of any palpable lymph nodes in the elbow region in both groups; the latter group exhibited epitrochlear palpable lymph nodes in 27% of cases [7]. This result highlights the importance of performing a routine epitrochlear region examination in patients who exhibit lymphadenopathy in any other area of the body.

In the clinical evaluation of our patient, there was a solid, nonmobile mass in the medial area of the right arm without skin alteration. Skin involvement is a rare occurrence and has been documented solely by Llamas-Velasco et al. in a case report involving a 44-year-old man with an enlarged epitrochlear node, erythematous indurate papules and plaques in the right upper limb. Histopathological analysis, both on the skin and on the lymph nodes, confirmed HL subtype–mixed cellularity [8]. Imaging is often utilized to complement the diagnostic process, particularly after physical examination of superficial lymph nodes. In this regard, US serve as the initial diagnostic approach, due to its rapid, cost-effective, safe, and efficient nature compared to other modalities. It allows assessment of morphology, echogenicity, dimensions (short axis), margins, number, and vascularization of lymph nodes. Normal lymph nodes usually appear as small ellipsoid or reniform formations, with a short axis < 5 mm, characterized by a hypoechoic cortex and a vascularized hyperechoic hilum [6]. On the contrary, malignant lymph nodes can appear large (short axis > 5 mm), irregular in shape, hypoechoic, with thickened cortex, with absent fatty hilum and altered blood flow.

Elbow region masses may have a nodal or extra-nodal origin. Nodal masses are represented by lymphadenitis of various etiology, such as acute bacteria, tubercular, sarcoidosis-related, due to foreign bodies or IV drug abuse, but also lymphomas or metastatic lymphadenopathies. Extra-nodal masses are represented by tumors (median nerve tumors, fibromas, hemangiomas, lipomas, Merkel cell tumors), sebaceous cysts, abscesses (elbow joint septic arthritis), Kimura disease, and cutaneous or subcutaneous hematogenous metastases [6, 9]. An unusual case of HL extra-nodal involvement of the elbow is reported in literature by Jawa et al. in a 33-year-old man affected by a synovial non-Hodgkin's B-cell lymphoma of the elbow presenting with joint contracture, loss of motion, pain, and intermittent paresthesia to the fourth and fifth fingers of the hand [10].

Currently only very limited information is available about patients with HL with epitrochlear lymph node involvement. This very rare presentation of Hodgkin disease was first described 1932 by Rouviere [4]. In the following years, some authors have reported epitrochlear lymph node positivity in rare, isolated cases or several large series of HL.

Landberg and Larsson have determined epitrochlear or popliteal lymph node involvement in only 4 of 149 patients with HL (2.6%). In contrast to our case, concurrent involvement of lymph nodes in other sites was observed in all instances [11]. Weiss and Jenkins describe a case of a 31-year-old man presenting with epitrochlear node involvement by mixed cellularity type HL as the primary and predominant manifestation, with a clinical stage CS IA and normal bone narrow aspirates, with findings similar to our patient. However, Weiss patient also demonstrated a single pathologic nodule in the spleen, with a subsequent pathologic stage IIIA [5].

Chang et al. studied 17 patients with HL and epitrochlear or brachial lymphadenopathy, with a median follow-up of 17 years [4]. Eleven patients had clinical stage IA-IIB, in line with our patient CS IA, while the remaining patients presented CS III-IV. The authors found that 82.3% of the patients were men. The median age at diagnosis was 27, lower than our patient. The majority (9 patients) showed an involvement of the right elbow, only one bilateral and the remaining 7 an involvement of the left one [4].

In Chang's study, nodular sclerosis was the most prevalent histology observed (41%), while lymphocyte-rich type of HL was reported at a frequency of 24% [4]. Due to the infrequent occurrence of isolated HL presenting with epitrochlear involvement, there is not a clear consensus on the optimal treatment approach.

Treatment strategies have given excellent results in Chang et al. series, in which 13 patients were treated with RT alone, 1 patient with CT alone (3 cycles of MOPP-mechlorethamine, vincristine, procarbazine, and prednisone—and 3 cycles of cyclophosphamide, vincristine, procarbazine, and prednisone) and 3 patients with RTCT (including even ABVD protocol-doxorubicin, bleomycin, vinblastine, and dacarbazine) [4].

A synergy of RT and CT was the treatment approach chose for our patient.

In conclusion, the scarcity of disease manifestation and symptomatology leads to a late diagnosis (2 years after the first appearance of tumefaction). Despite these challenges, a favorable therapeutic response was observed with a disease-free period of 4 years to date.

### 3.1. Differential Diagnosis of the Soft Tissue Malignant Neoplasm of the Elbow

Soft tissue neoplasms of the elbow constitute approximately < 5% of the entire spectrum of soft tissue tumors [12]. Predominantly, elbow soft tissue lesions demonstrate benign characteristics while the malignant tumors are extremely uncommon. The last include synovial sarcoma, myxofibrosarcoma, undifferentiated pleomorphic sarcoma, and MPNSTs [12].

Notably, the proximity of these tumors to neurovascular structures contributes to the onset of symptoms due to compressive effects, such as pain, swelling, functional limitation, alteration of sensitivity, and even stiffness, weakness, and skin changes.

Even though US can be performed, in the suspect of a malignancy in this region, the gold standard technique is the contrast-enhanced MRI, while biopsy is essential for histological confirmation. Total body CT is used to investigate the presence of potential metastasis, as well as FDG/PET-CT, is the main alternative to CT for the assessment of response to neoadjuvant therapy [12]. In literature, it has been observed that soft tissue malignant tumors of the elbow tend to metastasize primarily to the lung, followed by lymph nodes and bone [13].

The major challenge of this type of tumors is not only an early and accurate diagnosis but also a correct treatment, which should preserve as much upper extremity mobility as possible.

The main therapeutic goal is to preserve the function of the limbs and at the same time achieve effective control over the tumor. For superficial tumors or small deep tumors (< 5 cm) which do not involve critical structures, a wide excision with negative margins (ranging from 1 to 2 cm) might be enough [14, 15].

In case of tumors close to neurovascular structures or bone, positive margins might be present, necessitating RT to mitigate the risk of local recurrence [16]. The recurrence rate, in fact, is approximately 50%. It is a game of pros and cons, where preserving as much as possible the functionality of the limb must be put on balance with the risk of local recurrence and progression of disease [17].

The most common approach to reduce the local recurrence includes CT and RT. While the use of CT remains a subject of controversy, RT has proven to be a valuable tool, in particular as neoadjuvant therapy to reduce the risk of postsurgery local recurrence [18, 19].

#### 3.1.1. Synovial Sarcoma

Synovial sarcomas are malignant soft tissue tumors, intermediate-high grade, initially with a slow rate of growth that commonly affect young adults [20].

These tumors present as a painless and deeply situated mass, which affect the upper and lower extremities, with a specific predilection for the periarticular zones near major joints [12]. The incidence rate within the upper extremity falls within the range of 16%–25%. These masses appear as either well-defined or poorly defined heterogeneous structures, frequently heterogeneous due to the presence of necrosis, cystic degeneration, fibrosis, or calcifications [21].

The presence of extensive calcification is seen to be linked to long-term survival outcomes [22]. Limited cases of synovial sarcoma in the elbow have been documented in literature.

One of the earliest reported cases dates back to 1989, when Rinehart et al. followed the case of a 23-year-old girl who had intermittently pain from the elbow to the palm of her hand for about 2 years. Through palpation, the doctors identified a mass in the flexion crease of the elbow, associated with paresthesia in three fingers of the hand during the palpation. Histological examination confirmed the diagnosis of synovial sarcoma and the surgical excision of the mass followed by the reconstruction of the area resulted in favourable outcomes, with good residual functionality [23].

Although the precise etiology of this tumor remains unknown, a translocation between the X and 18 chromosomes [t(X; 18) (p11.2; q11.2)] [24].

Nevin and King presented the case of a 24-year-old female, with a history of progressive pain and stiffness of the elbow, without any associated inflammatory symptoms or history of local trauma. Following the identification of a cystic-like mass anterior to the trochlea at MRI examination, the patient underwent surgical intervention with a final histology revealing a synovial sarcoma, associated with SYT translocation of 18q11.2 [25].

The optimal therapeutic approach has not yet been determined, even though the preferred treatment involves complete surgical excision followed by adjuvant RT [26].

**Figure 5:**
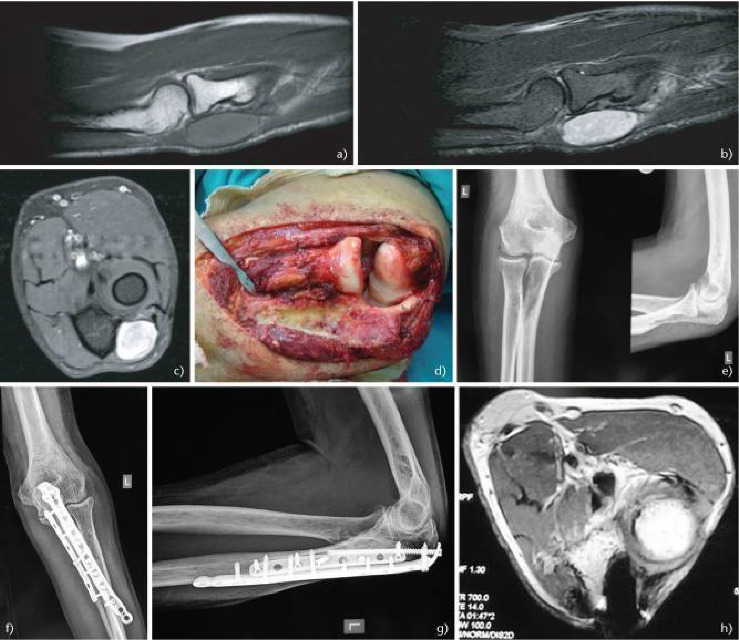
(a) Sagittal T1-weighted magnetic shows a mass with homogeneous intensity. (b) Sagittal T2 short tau inversion recovery (STIR). (c) Axial T1 fat saturated after contrast medium shows homogeneous enhancement of the lesion. (d) Intraoperative image after the wide excision of the synovial sarcoma. (e) Postoperative radiograph shows a pathological fracture in the proximal ulna due to radiation therapy and (f; g) its treatment after fixation with plates and screws. (h) Postoperative axial T1-weighted imaging after contrast medium shows no signs of recurrence 3 years after surgery.

Savvidou et al. shows the case of a 64-years-old man who presented a small mass on his left elbow which the surgical excision confirmed to be a synovial sarcoma. The patient underwent adjuvant RT, which led to a pathological fracture of the proximal ulna, surgically treated with plates and metal screws. The MRI performed 3 years later showed no signs of loco-regional recurrence, confirming the success of the treatment performed [12] (Figure 5).

Courtesy of Savvidou et al. [12].

#### 3.1.2. Myxofibrosarcoma

Myxofibrosarcomas are characterized by malignant fibroblastic growths surrounded by a myxoid stroma. They tend to manifest more often in upper extremities, with an elbow involvement of about 3%, more commonly presenting in elderly, male predominance [27].

Unlike most other histological variants of low-grade soft-tissue sarcoma, these tumors tend to exhibit an infiltrative nature, which probably explains the elevated rate of local recurrence, reported in recent studies (approximately 15%–20%) [28]. The infiltrative growth pattern of these tumors follows the fascial planes, and it is seen as a high signal “tail” on MRI [27].

Surgery represents the chosen treatment also in this disease, even though reconstruction or flap coverage should ideally be performed as a separate procedure, following the confirmation of negative margins. Additionally, neoadjuvant RT holds significant importance in the management [29].

The histological grading categorizes myxofibrosarcoma into grades from 1 to 3, based on factors such as tumor differentiation, mitotic count, and the presence of tumor necrosis [28].

Although the high rate of local recurrence and residual disease in these tumors, distant metastasis occurrence is actually very low when compared to other sarcomas [30]. When hematogenous spread occurs, it can affect various sites, altering the survival rate. In the most recent literature, there is a case of an 80-year-old woman who arrived at the Wake Forest University School of Medicine with a fungoid mass on her right elbow and left hip, which was revealed to be a high-grade myxosarcoma after biopsy was performed; the CT scan showed pulmonary metastases as well as suspected disease localizations in the right axilla and para-aortic region [31].

#### 3.1.3. Undifferentiated Pleomorphic Sarcoma

Undifferentiated pleomorphic sarcoma, previously known as Malignant Fibrous Histiocytoma [32], exhibits a highly aggressive biological characteristic and unfavorable prognosis.

In 20% of cases, originates in the upper limbs, particularly in the elbow region. Typically, it presents in elderly individuals as a slow-growing palpable mass without specific painful symptoms.

Usually, these tumors are well-circumscribed and are situated either within or near muscle tissues. In MRI, typically appear with a heterogeneous signal, due to the presence of calcifications, areas of necrosis, or hemorrhage, with an enhancement of the solid components [33].

Treatment strategies are based on CT and RT, in addition to surgical intervention.

Long-term studies have demonstrated that resection of the mass, performed with wide margins and CT, did not negatively impact survival or local control compared to amputation [34].

Alok Chandra Agrawal reported an interesting case of a 32-year-old woman with history of pain and swelling of her left elbow for about 15 days. The X-ray performed upon access showed extensive osteolytic changes in the ulna.

The biopsy confirmed the presence of a malignant fibrous histiocytoma of the bone (previous name of the undifferentiated pleomorphic sarcoma). These demonstrated that, although these tumors are generally classified as soft tissue lesions, in some cases, they may arise in the bone.

Initially, an amputation of the limb had been proposed to the patient; subsequently, the execution of an US showed the absence of vascular infiltration by the mass, so it was opted for a less invasive surgery.

The subsequent reconstruction surgery allowed the patient to preserve good elbow and hand function with a disease-free follow up to 10 years [35].

#### 3.1.4. MPNST

MPNSTs are rare and aggressive soft tissue sarcomas that originate from peripheral nerves or their sheaths; they can emerge either independently or as a transformation from pre-existing tumors like neurofibromas or schwannomas. Around 50% of these tumors are identified in individuals with neurofibromatosis type I (NF1) [36]. Treatment for MPNSTs typically includes a combination of surgery, radiation therapy, and sometimes CT.

The patient's health, tumor size, location, grade, and extensions are the main factors that influence prognosis.

**Figure 6:**
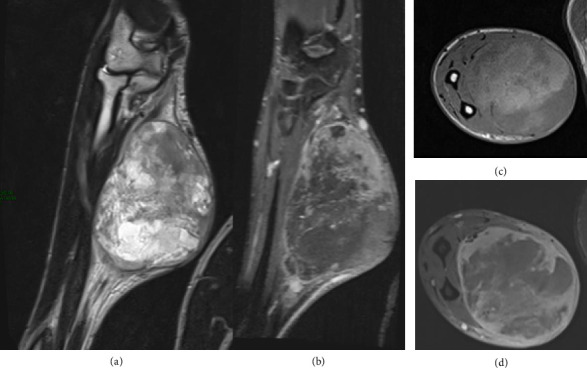
MRI images. A well-defined solid mass with regular margins located in the middle third of the medial compartment of the forearm. The lesion appears to originate along the course of the ulnar nerve, not clearly recognizable, involving the flexor muscles, both intra and interfascial. A thin plane of cleavage from the ulnar diaphysis is preserved. (a) Coronal T2 TSE; heterogeneous with a prevalent hyperintense component, due to a fluid component. (b) Coronal T1 postcontrast; the lesion shows heterogeneous postcontrast enhancement. (c) Axial T1 TSE; (d) Axial T1 postcontrast.

Prognosis can be positively impacted by early diagnosis, complete surgical resection with clear margins, and effective adjuvant therapies [37]. In our hospital, we treated the case of an 18-year-old male with NF1, who developed a tumor of the peripheral nerve sheaths of the elbow. The young man presented to our emergency department upon observing the appearance of a swelling in the right forearm. This swelling had progressively increased in size over the past months and was accompanied by the onset of pain and functional limitation of the ipsilateral hand. A contrast-enhanced MRI of the arm was performed (Figure 6), revealing suspicious alterations suggestive of malignant degeneration of a neurofibroma/schwannoma.

Distinguishing between a benign neurofibroma and schwannoma is generally considered challenging based on imaging criteria. Schwannomas are benign tumors that arise from Schwann cells. Neurofibromas are also benign tumors but they originate from a mixture of different cell types including Schwann cells, fibroblasts, and perineural cells.

Schwannomas are encapsulated and manifest as hyperintense on T2-weighted imaging due to their elevated water content and present homogeneous enhancement. Neurofibromas have a strong heterogeneity owing to the presence of different cell types within the tumor. Although neurofibromas may present a less intense enhancement after the administration of intravenous medium contrast.

A core biopsy of the mass of the young patient exhibited histological features indicative of a fusiform cell sarcoma, highly suggestive of MPNSTs with epithelial differentiation.

Because of the disease extension at the time of diagnosis, a multidisciplinary team opted to start a neoadjuvant CT, which was prematurely interrupted due to the onset of complications. Hence, the subsequent surgical intervention did not achieve a radical outcome and residual/recurrence of disease in the following months led to a significant disease progression with the appearance of lung metastases. All of this resulted in an unfortunate outcome for the young patient.

## 4. Conclusion

Malignancies localized in the elbow region are a rare medical phenomenon. This holds true for solid tumors, such as synovial sarcoma, myxofibrosarcoma, undifferentiated pleomorphic sarcoma, and MPNSTs. It is an even rarer scenario in the case of hematological tumors, as underscored by our singular presentation of HL in the elbow.

Since a prompt and precise diagnosis represents the cornerstone for enhancing patient prognosis and longevity, a meticulous clinical examination of the elbow becomes paramount for medical professionals, especially when patients present with related complaints. Additionally, in these instances, imaging, complemented by a biopsy when warranted, becomes fundamental.

Our case aims to underscore the vast spectrum of HL presentations, challenging our conventional diagnostic paradigms and suggesting a holistic and tailored approach to patient care.

## Data Availability

The data that support the findings of this study are available from the corresponding author upon reasonable request.
